# Sustained Virologic Suppression Reduces HIV-1 DNA Proviral Levels and HIV Antibodies in Perinatally HIV-Infected Children Followed from Birth

**DOI:** 10.3390/v14112350

**Published:** 2022-10-26

**Authors:** Trevon Fuller, Tara Kerin, Ruth Cortado, Maria de Lourdes Benamor Teixeira, Maria Isabel Fragoso da Silveira Gouvêa, Christianne Moreira, Maria Leticia Santos Cruz, José Henrique Pilotto, Ivete Gomes, Breno Santos, Tauí Rocha, Priya R. Soni, Esau Joao, Myung Shin-Sim, Yvonne Bryson, Karin Nielsen-Saines

**Affiliations:** 1 Institute of the Environment & Sustainability, University of California Los Angeles, Los Angeles, CA 90095, USA; 2 Infectious Diseases Department, Hospital Federal dos Servidores do Estado, Rio de Janeiro 20221-161, RJ, Brazil; 3 Pediatric Infectious Diseases, David Geffen UCLA School of Medicine, Los Angeles, CA 90095, USA; 4 Instituto Nacional de Infectologia Evandro Chagas (INI), Fundação Oswaldo Cruz (FIOCRUZ), Rio de Janeiro 21040-360, RJ, Brazil; 5 Hospital Geral de Nova Iguaçu, Nova Iguaçu 26030-380, RJ, Brazil; 6 Laboratório de AIDS e Imunologia Molecular, Instituto Oswaldo Cruz (IOC), Fundação Oswaldo Cruz, (FIOCRUZ), Rio de Janeiro 21040-360, RJ, Brazil; 7 Hospital Nossa Senhora da Conceição, Porto Alegre 91350-250, RS, Brazil; 8 Cedars Sinai Medical Center, Los Angeles, CA 90048, USA

**Keywords:** early HIV treatment, viral reservoir burden, HIV DNA droplet digital PCR, viral load kinetics, perinatal HIV infection

## Abstract

The extent to which perinatally HIV-infected children, following cART initiation, develop a low proviral reservoir burden over time, as measured by HIV DNA droplet-digital polymerase chain reaction (ddPCR) and the effect on HIV antibody is not well characterized. We measured proviral HIV DNA and plasma RNA virus load (VL) in 37 perinatally HIV-infected children at 6 months of age who initiated stable cART. At 6–11 years of age, HIV proviral DNA, HIV VL (RNA), and HIV antibody by Western Blot (WB) were assessed. CART was initiated before 6 months of age in 13 children and after 6 months in 24. At school age, the HIV DNA levels did not differ by the timing of cART, and the HIV DNA levels were lower in children with negative/indeterminate WB (*p* = 0.0256). Children with undetectable HIV RNA VL > 50% of the time since cART initiation had lower median DNA VL than children with undetectable VL < 50% of the time (*p* = 0.07). Long-term viral suppression in perinatally HIV-infected children is associated with a decrease in HIV antibodies and reduced HIV reservoirs.

## 1. Introduction

Currently, 1.7 million children under 15 years of age are living with HIV around the world [1]. Previous studies investigated associations between the timing of initiation of combination antiretroviral therapy (cART) and virologic and immunological endpoints in childhood [2,3,4]. Early in infection, HIV provirus integrates into reservoirs such as central memory T cells [5,6]. Studies have demonstrated that the early initiation of cART impacts HIV reservoir size [7,8,9]. Infants who acquire HIV perinatally and are treated with cART in the first year of life have significantly lower levels of HIV proviral reservoirs in early childhood than those initiating cART after 1 year of age [10,11]. The risk of HIV-disease morbidity and mortality is also significantly higher in infants subject to deferred initiation of cART [12]. Therefore, the present guidelines for the treatment of perinatal HIV infection call for the initiation of combined antiretroviral therapy as early as possible following diagnosis [13,14]. 

There is scant data examining how the duration and timing of cART initiation in early childhood may affect viral dynamics and immune biomarkers in school-aged children up to early adolescence. The viral kinetics of early HIV infection in infants, including the magnitude and suppression of viremia, can potentially be distinct depending on the timing of HIV infection (in utero/ intrapartum) and the type of prophylactic antiretroviral regimen used postnatally to prevent HIV acquisition [15]. Among intrapartum HIV-infected infants, the timing of early diagnosis (i.e., first positive DNA or RNA HIV test) could be influenced by the potency of the antiretroviral regimen used for HIV prophylaxis in the neonatal period [16]. In addition, varying degrees of viral suppression during early infection may influence CD4 count fluctuations and disease outcomes over time. Early treatment preserves immune function and avoids CD4 cell count depletion. The objective of the present study was to compare virologic and immunological biomarkers in a cohort of perinatally HIV-infected children recruited at three HIV Prevention of Mother to Child HIV Transmission (PMTCT) centers in Brazil since the time of birth. 

## 2. Materials and Methods

Early pediatric cohort: The cohort was comprised of HIV-infected children originally enrolled in a separate prospective, phase III, open-label, randomized clinical trial (NICHD HTPN 040 study) conducted between 2004 and 2010 [17,18]. This trial was developed for women who did not receive antiretrovirals (ARV) during pregnancy because they were not identified until the time of labor and delivery. The infants were enrolled within 48 h of birth and randomized to 3 different treatment arms (ZDV for 6 weeks monotherapy; ZDV for 6 weeks plus 3 doses of nevirapine (NVP); or ZDV for 6 weeks plus 2 weeks of nelfinavir (NFV)+ lamivudine (3TC)) for HIV PMTCT. The timing of HIV infection was determined as in utero or intrapartum based on the timing of a positive DNA PCR test following birth [19]. All of the infants were formula-fed. The data collected from each infant at birth included HIV RNA plasma viral load (VL) copies/mL based on quantitative PCR (Abbott Realtime, Abbott Molecular, Inc., Des Plaines, IL, USA), 4th generation HIV ELISA, CD4+, and CD8+ T cell counts measured by three-color flow (TruCOUNT, FACSCalibur system). Each infant was followed until 6 months of age with HIV infection status primary endpoint determined at 3 months. Following study completion, the participants in the present cohort had HIV RNA VL measurements performed at a minimum yearly using quantitative HIV RNA PCR as per standard of care. The lower limit of detection of the quantitative HIV RNA VL was 40 copies/mL of plasma.

Older pediatric cohort: The original participants of the perinatal trial were recruited into the present cohort. All of the children had an established diagnosis of perinatal HIV infection, determined by at least 2 HIV RNA measures above the threshold of detection at two different timepoints. The children were recruited from 3 HIV PMTCT centers, which had participated in the original perinatal trial in Brazil [18]. To be recruited, children needed to have been in medical follow-up with written parental/guardian informed consent obtained. Recruitment occurred in 2015–2017; the children were 6–11 years of age at the time of enrollment. 

Study procedures: The present study was a prospective cohort study with 2 visits 6 months apart between 2015 and 2017. During the first visit, clinical, laboratory, and social and demographic data were collected. The serum samples were collected and tested via BioRad Genscreen (GS) HIV-1 Fourth Generation ELISA Kit (Biorad, Hercules, CA, USA). HIV Western Blots (Biorad, Hercules, CA, USA) were performed and classified as per CDC guidelines. CD4+ and CD8+ T cell percentage, absolute counts, and quantitative HIV RNA VL, measured as copies/mL were determined. During the second visit, peripheral blood mononuclear cells (PMBCs) to measure HIV DNA VL by ddPCR were collected. ddPCR is a very sensitive PCR platform that enables absolute quantification of a DNA target molecule, thus quantifying total HIV DNA in PBMCs as a measure of HIV viral reservoirs [20,21]. In the present analysis, full-length viral DNA was measured at the HIV Gag/LTR junction [22,23].

Combination antiretroviral regimens and medical follow-up: cART was administered based on Brazilian Ministry of Health Guidelines as per standard of care [24]. cART was dispensed free of charge per the Brazilian Single Unified Health System, with prescriptions verified by hospital pharmacies [25]. The age of cART initiation was abstracted from the medical records. For the purposes of this study, early cART was defined as ART initiated up to 6 months of age, and late cART as ART initiated after 6 months of age. We assembled data on each child’s cART regimen(s) and those who switched to a second-line regimen, which was defined as changing at least 1 NRTI and changing the backbone drug class or changing the protease inhibitor [26]. Response to cART was defined as follows: 

Response: Undetectable plasma VL at study enrollment, with HIV RNA VL undetectable more than 75% of the time since cART initiation earlier in childhood; 

Partial response: Undetectable plasma VL at study entry and undetectable HIV RNA VL between 50–75% of the time since ART initiation earlier in childhood; 

Non-response: Detectable plasma HIV RNA VL at study entry and/or HIV RNA VL detectable more than 50% of the time since cART initiation earlier in childhood. 

Partial responders and responders were combined for analysis of viral reservoirs due to sample size. Differences in means were compared using Welch’s *t*-test for normally distributed variables and the Mann–Whitney test for variables that were not normally distributed. The log-rank test was used to compare the average number of months of cART use until the first undetectable RNA VL in children who initiated cART at less than 6 months of age versus those who started cART later. Analyses were performed with Stata v. 17 and PRISM v. 8. The parents/guardians provided written informed consent, and the study was approved by the local Institutional Review Boards (approval number: CAAE 40856115.5.1001.5252).

## 3. Results

**Figure 1:**
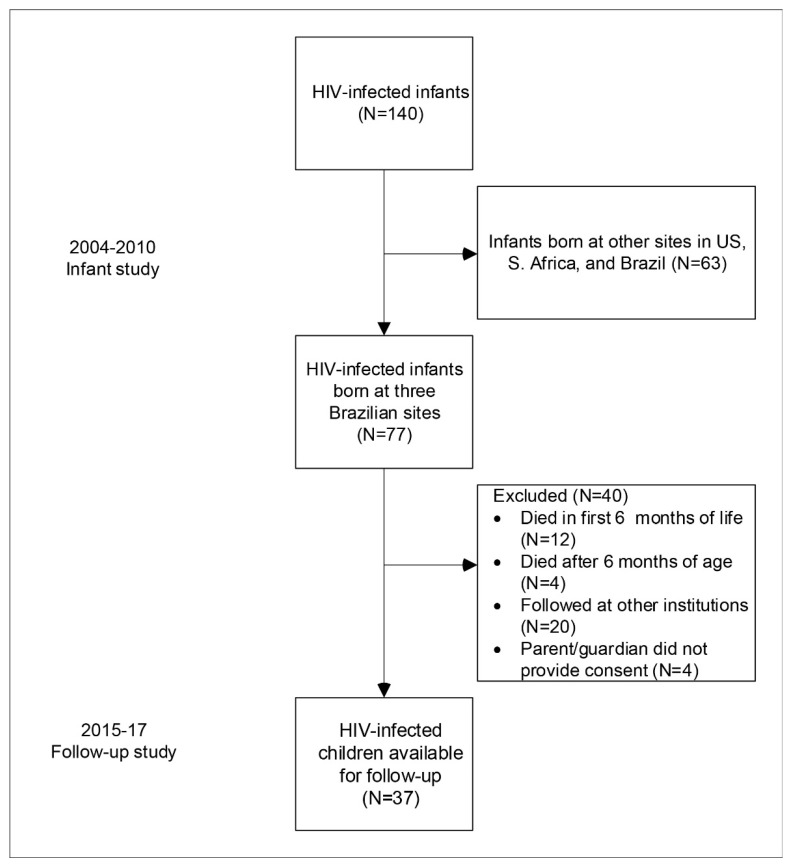
Flowchart of children living with HIV enrolled in the present study.

A total of 140 perinatally HIV-infected children were identified in the original perinatal cohort of 1684 HIV-exposed infants (8.3%) (Figure 1). Over half of these children (*n* = 77) were recruited at the three Brazilian sites participating in the current study (two sites in Rio de Janeiro and one in Porto Alegre). Among 77 children, 12 (16%) died within the first 6 months of life while still in the original study, mainly due to infectious causes such as sepsis, pneumonia, gastroenteritis, encephalitis, and also sudden infant death syndrome (SIDS), and an additional 28 were excluded, as shown in Figure 1. Among those remaining, 23 of 37 (62.2%) were determined to be HIV-infected in utero, and the remaining 14 were intrapartum. Seventeen of thirty-seven infants (46%) tested positive for syphilis at birth (Table S1). 

The median age of initial cART initiation for HIV treatment purposes was 8 months (IQR: 5–19.5). Thirteen (35.1%) children initiated the use of cART early, and 24 (64.9%) started late. In total, 46.2% (6/13) of the children who initiated cART early and 58.3% (14/24) who initiated cART late were females (Table S1). With respect to the mode of delivery, 6/13 (46.2%) of the children who started cART early were born by spontaneous vaginal delivery versus 66.7% (16/24) of the children who started cART late. In total, 92.3% (12/13) of the infants who started cART early acquired HIV via in utero transmission compared to 45.8% (11/24) of the children who started cART late. 

**Figure 2:**
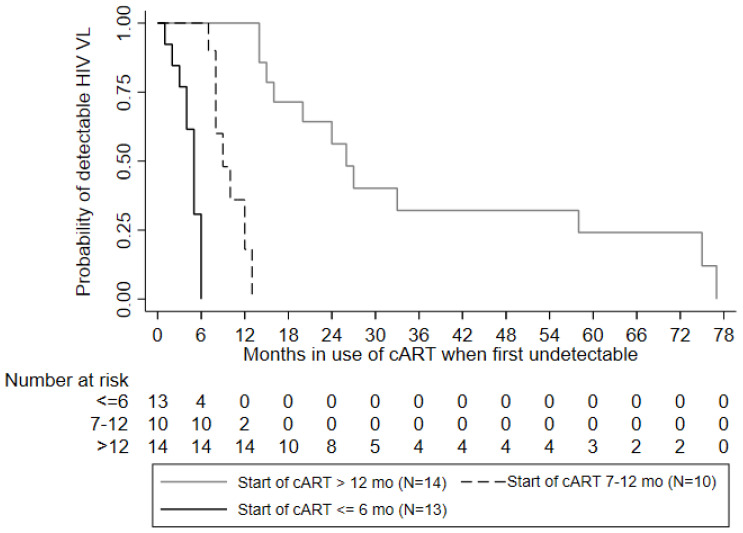
Kaplan–Meier survival curves indicating the effect of cART initiation on time to virologic suppression of HIV RNA (N = 37). In total 13 infants initiated cART at up to 6 months of life, and 24 after 6 months. There were 4 children who never achieved undetectable VL after starting cART.

From birth (within 48 h of delivery) to 6–11 years of age, the mean number of RNA VL tests was 14 for children who initiated cART early and 15 for those who started late. For children who started cART by 6 months of age, the median number of months required to achieve an undetectable viral load was five, whereas for children who started between seven and twelve months, the median number was ten months. The number of months to achieve undetectable viral load after starting cART differed significantly by the age of cART initiation (log-rank test *p* < 0.0001). The maximum number of months required to achieve undetectable viral load for children who began cART by 6 months of age was six months, whereas, for children who began cART between seven and twelve months of age, the maximum was 13 months (Figure 2). 

**Figure 3:**
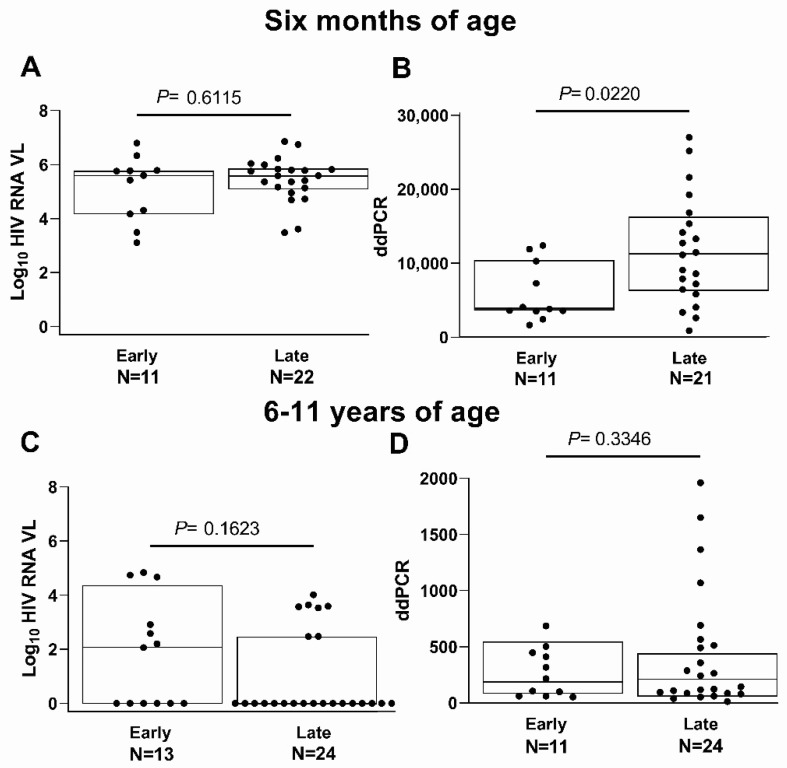
Virologic and immunologic endpoints by time of treatment initiation. (**A**) HIV RNA VL at 6 months of age (N = 33); (**B**) HIV DNA ddPCR at 6 months of age (N = 32); (**C**) HIV RNA VL at 6–11 years of age (N = 37); (**D**) HIV DNA ddPCR at 6–11 years of age (N = 35).

At 6 months of age, the HIV RNA VL results were available for 33 of 37 children, 84.6% (11/13) of whom started cART early and 91.7% (22/24) late. Infants who initiated cART early had a median log_10_ HIV RNA VL of 5.6 copies/mL; the median VL was also 5.6 copies/mL at 6 months for children initiating cART late (*p* = 0.61) (Figure 3A). Thirty-two children had ddPCR levels performed at 6 months of age; 84.6% (11/13) started cART early, and 87.5% (21/24) late. At 6 months of age, infants who started cART early had a median HIV DNA ddPCR of 3086 copies/million PBMCs versus 11,138 per million PBMCs for those who started late (*p* = 0.02) (Figure 3B). Assays for HIV antibody by WB were not performed under 18 months since they only reflect maternal antibody. 

At 6–11 years of age, all 37 children had multiple HIV RNA VL results available over time. Table S4 lists the children’s cART regimens and those who switched regimens. The median HIV RNA VL at school age of children who started cART early was 2.0 log_10_ copies/mL; six children who started early had an undetectable VL at this time point. Of the 24 who started late, 17 had undetectable RNA VL at school age. The seven with detectable VL had a median VL of log_10_ 3.6 copies/mL at school age (Figure 3C). At this later time point, 94.6% (35/37) of children had ddPCR results available, 84.6% (11/13) of those who started cART early, and 100% (24/24) who started late. The median HIV DNA ddPCR of the children who started cART early was 193 copies per million PBMCs versus 215 for those who started late (*p* = 0.74) (Figure 3D). 

At the time of the study visit for children returning at 6–11 years of age, all of the participants were tested for HIV-1/2 antibodies by EIA and were found to have a positive serology result. The WB results were available for 36 children, 9 of 12 (75%) early-treated children and 19 of 24 (79.2%) late-treated children had positive WB results. The remaining eight children had negative or indeterminate WB results. Median VL results and ddPCR results at 6 months and at 6 to 11 years of age did not differ by type of neonatal prophylaxis regimen, nor did the WB results at 6 to 11 years of age (Tables S2–S4).

**Figure 4:**
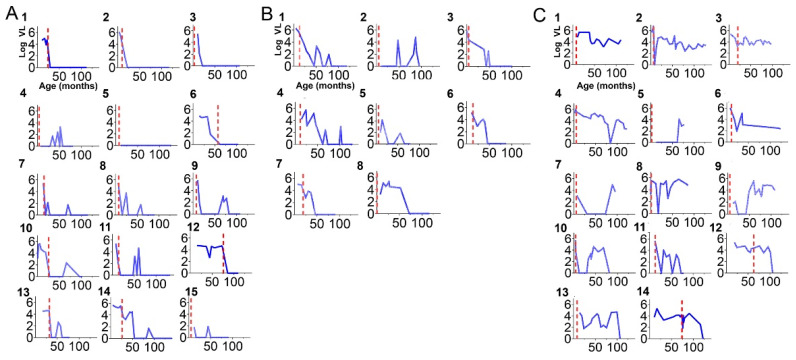
HIV RNA viral load kinetics according to response to therapy status. (**A**) Complete responders (N = 15) children with an undetectable VL at study entry with HIV RNA viral loads undetectable more than 75% of the time since cART initiation in early childhood. The dashed red line depicts the child’s age at cART initiation. (**B**) Partial Responders (N = 8), children with an undetectable VL at study entry with undetectable HIV RNA VL between 50–75% of the time since cART initiation in early childhood. (**C**) Non-responders (N = 14), children with detectable HIV RNA VL at study entry and/or HIV RNA VL detectable up to 50% of the time since cART initiation in early childhood.

At the time of study entry, 62.2% (23/37) of children were responders or partial responders to cART, whereas 37.8% (14/37) were non-responders (Figure 4A–C).

**Figure 5:**
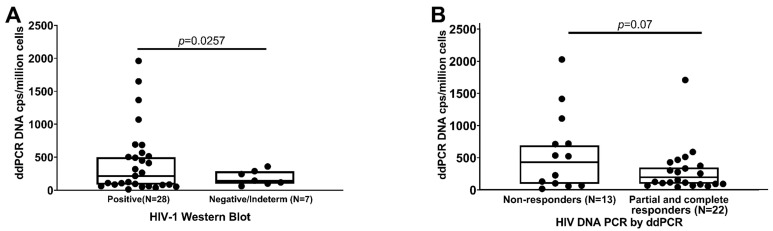
Viral reservoirs during childhood (6–11 years of age) measured by HIV DNA ddPCR. (**A**) HIV DNA ddPCR according to HIV-1 Western blot results; (**B**) HIV DNA ddPCR results according to response to cART; One child classified as a responder based on HIV RNA did not have ddPCR available, and 1 classified by HIV RNA as a non-responder did not have ddPCR.

The median HIV DNA VL of the WB-positive children by ddPCR was 215 copies/million PBMCs versus 144 copies for those with negative/indeterminate WB results (*p* = 0.0257) (Figure 5A). One child with a WB result did not have a corresponding ddPCR result, and this child had a negative/indeterminate WB. The ddPCR results were available for 22 of 23 responders and partial responders and 13 of 14 non-responders. Partial responders and responders had a mean HIV DNA ddPCR of 109 copies/million PBMCs, whereas non-responders had higher HIV DNA ddPCR levels of 602 copies/million PBMCs (*p* = 0.07) (Figure 5B).

## 4. Discussion

In the present study, the maintenance of an undetectable HIV RNA VL correlated with a lower HIV DNA reservoir size and the loss of HIV-1 specific antibodies over time. Another important contributor to the pathophysiology of viral reservoir burden is possibly the role of viral blips in the seeding of viral reservoirs [22]. Even among responders, only five children maintained consistently undetectable virus loads after the initiation of cART (Table S5). Maintaining consistently undetectable virus loads after the initiation of cART is probably the most important mechanism through which a reduced HIV reservoir burden is achieved. If children start cART early, but the maintenance of an undetectable VL throughout childhood is not attainable, the HIV DNA viral reservoir burden will increase. In the present study, children with undetectable RNA VL more than half of the time since cART initiation had lower median DNA VL than children with undetectable VL less than half of the time. The observed relationship between RNA VL and the HIV DNA viral reservoir suggests that suppressive ARV treatment during early childhood can have a greater effect on reducing the HIV reservoir than initiation of treatment later in chronic infection. HIV RNA levels are more dynamic and susceptible to change than HIV DNA reservoirs, but even so, children might be able to have reduced viral reservoirs once viremia is controlled, even after an early course where viremic control was not immediately achieved. 

Studies of early-treated HIV-infected infants and older individuals have demonstrated that when patients receive cART shortly after initial HIV infection, HIV antibodies might not develop [11,27,28]. Nearly one-third of perinatally infected children had a negative or indeterminate WB result later in life; this was associated with a significantly smaller viral reservoir size. Our findings suggest that cART treatment was associated with a seronegative WB result after several years of cART use. Furthermore, some children in our study, despite starting cART late, achieved and maintained an undetectable HIV RNA VL, and had a seronegative or indeterminate HIV WB at 6–11 years of age. Perhaps this could suggest that reservoir size in children can still be reduced even with delayed cART initiation, provided that the child achieves long-term suppression of HIV RNA VL. Lack of HIV antibodies can be useful as a surrogate screening tool to identify children with low HIV reservoir burden. This is similar to a previous study showing that children who initiated cART in infancy and remained cART adherent for 2 years experienced a decline in HIV viral reservoir size [29].

To date, only a small number of studies have assessed changes in proviral DNA levels over time in children [30]. In a study of 12 children starting cART at a median of 1.9 months and maintaining suppression for 5.5 years, recovery of replication-competent virus was possible in 84% of subjects, indicating that treatment did not prevent the establishment of latent provirus but significantly limited the size of HIV-1 viral reservoirs [31]. In a cross-sectional study of 97 children in Mali with virologic suppression, the median total HIV DNA level was 445 copies/10^6^ cells [32], which is slightly higher than the HIV DNA levels found in our children on follow-up (193 copies/10^6^ cells in the early treated cohort at 6–11 years of age and 215 copies/10^6^ cells in the late treated cohort in the same age group). This finding can likely be explained by the fact that children in the Mali cohort were younger. In a study of European children treated at a median age of 2.3 months, after 8 months of therapy, virologically suppressed children had HIV DNA levels of 43 copies/ 10^6^ cells [33], which is evidence of significant suppression of viral reservoirs following very early treatment, with a smaller viral reservoir size than what we observed in our cohort, likely an effect of earlier ART initiation. Similarly, in a cross-sectional study from Thailand, the median total HIV DNA of 15 children with virologic suppression at a median age of 6.3 years was 132 copies/10^6^ cells, which resembles HIV DNA levels observed in our early treated group of children [34]. Another study of 37 children demonstrated the quicker decay of viral reservoirs among children who rapidly achieved virologic suppression, i.e., by 1.5 years of age [35]. A study of 23 perinatally infected children also showed that children initiating treatment within the first 12 weeks of life had a proviral DNA reservoir six-fold smaller than children initiating ART beyond that age [36]. In a study from Canada of 12 vertically infected children, the initiation of ART within 72 h of life was significantly associated with HIV-1 reservoir decay, to the point that HIV-1 DNA was not detectable in enriched CD4 cells from four children [28]. In a follow-up study of 69 HIV+ children with sustained virologic suppression for 5 years or more, viral reservoir size was associated with the age of ART initiation, age when virologic suppression was achieved, and inversely correlated with the proportion of life spent on effective cART [37]. 

When the study participants were recruited during infancy for a perinatal study, international treatment guidelines did not recommend cART treatment for newborns without evidence of symptoms or a significant drop in CD4 cell counts. Despite successful enrollment in a clinical trial and the early diagnosis of HIV infection, this high-risk population of infants was challenging to maintain beyond the initial study period, with a high rate of mortality and loss to follow-up. Few participants had a complete response to therapy, and a very high proportion had viral blips or did not respond to therapy at all. Another important consideration that demonstrates the vulnerability of this population is that 46% of the infants had a diagnosis of congenital syphilis, 27% had congenital CMV, and an additional 35% and 24%, respectively, were born to mothers who had CT/NG infections. This perinatal cohort was highly vulnerable and born to women who were not diagnosed with HIV during pregnancy, with a very high frequency of sexually transmitted infections (STIs) [17,38,39,40,41,42]. These children would likely be at risk for a high HIV viral burden driven by the high prevalence of congenital syphilis and other co-infections in the absence of prompt initiation of cART, which puts them at an even higher risk of seeding viral reservoirs. Although some early studies have shown that infants infected in utero have a higher chance of disease progression than intrapartum infected infants [43], we did not observe associations between the timing of HIV infection in this group of patients (62% were infected in utero and 38% intrapartum) and virus load kinetics or viral reservoir size (data not shown). Our findings underscore that the treatment of HIV-infected infants is challenging and demands resources, attention, and diligent follow-up in order to be successful. We found that when children drop out of treatment, the benefit of early treatment is lost, and viral reservoirs are reseeded. 

Among the strengths of this study is that the participants came from a large, randomized study, and the characterization of HIV DNA reservoirs has been little studied in this population. A previous study of children in the US found that those who achieved virologic suppression before their first birthday and maintained optimal adherence had lower HIV DNA levels than those achieving virologic suppression later [11]. Our study population comes from low-resource communities in Brazil thar face challenges obtaining support from healthcare services and caregivers. The population was a homogenous group insofar as clinical care was provided at HIV-referral institutions that adhere rigorously to Brazilian national HIV-treatment guidelines 

The weaknesses of the study include the limited sample size and the fact that the viral load data were abstracted yearly after 12 months, so additional viral blips could have been missed, which would affect the viral kinetics, particularly in the responder group. Furthermore, during the early pediatric cohort, national guidelines called for ART initiation based on clinical and immunological criteria. It was recommended that children with moderate to severe symptoms and low CD4 counts based on the CDC classification system begin cART immediately, but cART was not recommended for children with milder symptoms and higher CD4 counts. As such, the children in this study with poorer clinical and immunological conditions were likely to have been prescribed cART earlier. In light of this, clinical and immunological conditions may have confounded the effect of age at cART initiation. Additionally, when the study was designed, it was decided that early treatment would be defined as any initiation of cART before 6 months of life. However, current guidelines in Brazil, the US, and Europe call for initiation of cART as soon as possible after diagnosis [13,14,24], which in practice would be much sooner than 6 months. As such, as noted above, the term “early”, as it is used in the present study, is a historical classification that differs from the current treatment guidelines. Nevertheless, ours is a pilot study applicable to other countries where HIV vertical transmission is still ongoing. Although the guidelines have changed and children receive treatment much earlier, there are unfortunately numerous instances where therapy may not be promptly initiated. The results from our study demonstrate that even in situations where ART may not be promptly initiated, there may still be favorable outcomes of ART on the reduction in the HIV viral reservoir burden, as long as sustained viral suppression is achieved. HIV-1 specific antibody seems to be associated with viral reservoir persistence and can be used to gauge the size of residual latent viral reservoirs in children on ART. 

Unfortunately, a considerable proportion of the children from the antenatal study died, were lost to follow-up, or were relocated to other institutions. Potentially the high prevalence of co-infections could be a contributor to high HIV viral load and viral reservoirs. Our cohort, nevertheless, is highly representative of the real-life scenario of children perinatally infected with HIV in Brazil. 

## 5. Conclusions

In conclusion, among perinatally HIV-infected children retained in care, long-term virologic suppression was associated with lower HIV DNA proviral reservoirs and the loss of HIV-1 specific antibodies. Further studies with larger sample sizes and longer durations and frequencies of follow-up may help clarify the role and kinetics of HIV reservoir dynamics in children.

## Data Availability

Scripts and de-identified data will be made available on GitHub.

## Supplementary Materials

The following supporting information can be downloaded at: https://www.mdpi.com/article/10.3390/v14112350/s1. Table S1. Baseline sociodemographic, immunological, and virologic characteristics of children living with HIV in Brazil. Table S2. Mode of perinatal HIV acquisition, virologic and immunological outcomes, and neonatal prophylaxis regimen by timing of cART initiation among the study participants. Table S3. Mode of perinatal HIV acquisition, virologic and immunological endpoints, timing of treatment initiation, and neonatal prophylaxis regimen by response to cART among the study participants. Table S4. Lifetime ART regimens for study participants. Table S5. Clinical and laboratory details for five children with sustained virologic response.
