# Supplementary material for: Sustained Virologic Suppression Reduces HIV-1 DNA Proviral Levels and HIV Antibodies in Perinatally HIV-Infected Children Followed from Birth
Source: Viruses. 2022 Oct 26;14(11):2350. doi: 10.3390/v14112350 (PMC9693172; doi:10.3390/v14112350)
Supplement: Supplementary file 1 [file viruses-14-02350-s001.zip › viruses-1895804-supplementary.pdf]

Table S1. Baseline sociodemographic, immunological, and virologic characteristics of children living with HIV in Brazil (N = 37).

	Median	IQR
Age in months at recruitment for follow-up study (N = 37)	101	85–112
Age at cART initiation (months) (N = 37)	8	Range: 0–76
Gestational age at delivery (weeks) (N = 37)	39	38–40
Log_{10} HIV RNA copies/mL at 6 months of age (N = 37)	5.59	4.96–5.82
CD4+ T cell count at delivery (N = 35)	1978	1256–2206
CD8+ T cell count at delivery (N = 35)	1108	764–1397
	n	**%**
Sex		
Male	17	45.9%
Female	20	54.1%
Race/Ethnicity		
Black	13	35.1%
Mixed	15	40.5%
White	9	24.3%
HIV Infection		
In utero	23	62.2%
Intrapartum	14	37.8%
	n	**%**
Mode of Delivery		
Cesarean	16	43.2%
Vaginal	21	56.8%
Co-infections at birth		
Infant VDRL + at delivery	17	45.9%
Infant CMV + at delivery	10	27.0%
Mother CT + at delivery	13	35.1%
Mother NG + at delivery	9	24.3%
HPTN NICHD 040 study arm:		
ZDV	19	51.4%
ZDV + NVP	8	21.6%
ZDV + 3TC + NFV	10	27.0%

VDRL: Venereal Disease Research Laboratory, CMV: Cytomegalovirus, CT/ NG: Chlamydia trachomatis and Neisseria gonorrhea, ZDV: Zidovudine, 3TC: Lamivudine, NFV: Nelfinavir; NVP: Nevirapine.

Table S2. Mode of perinatal HIV acquisition, virologic and immunological outcomes, and neonatal prophylaxis regimen by timing of cART initiation among the study participants (N = 37).

	**Timing of cART Initiation**	
	Early (N = 13)	Late (N = 24)
Mode of perinatal HIV acquisition		
In utero	12 (92.3%)	11 (45.8%)
Intrapartum	1 (7.7%)	13 (54.2%)
Neonatal prophylaxis regimen		
ZDV, N(%)	5 (38.5%)	14 (58.3%)
ZDV + NVP, N(%)	5 (38.5%)	3 (12.5%)
ZDV + 3TC + NFV, N(%)	3 (23.1%)	7 (29.2%)
Virologic and immunological endpoints		
Median log10 HIV RNA copies/mL at 6 months	5.4 (3.3–5.8)	5.5 (4.8–5.8)
Median ddPCR at 6 months of age (IQR), N = 32	3931 (3268–10,695)	9084 (4070–15,363)
Median ddPCR at 6–11 years of age (IQR), N = 35	105 (57–429)	193 (86–553)
Indeterminate/negative WB at 6–11 years of age, N(%)	3 (23.1%)	5 (20.8%)

IQR = interquartile range. Early refers to cART initiation up to 6 months of age, and late after 6 months.

Table S3. Mode of perinatal HIV acquisition, virologic and immunological endpoints, timing of treatment initiation, and neonatal prophylaxis regimen by response to cART among the study participants (N = 37).

	**Response to cART**		
	Responder(N = 15)	Partial (N = 8)	Non-responder (N = 14)
Mode of perinatal HIV acquisition			
In utero	7 (46.7%)	7 (87.5%)	9 (64.3%)
Intrapartum	8 (53.3%)	1 (12.5%)	5 (35.7%)
Neonatal prophylaxis regimen			
ZDV	11 (73.3%)	2 (25%)	6 (42.9%)
ZDV + NVP	2 (13.3%)	1 (12.5%)	5 (35.7%)
ZDV + 3TC + NFV	2 (13.3%)	5 (62.5%)	3 (21.4%)
Timing of cART initiation			
Early treatment	3 (20%)	4 (50%)	6 (42.9%)
Late treatment	12 (80%)	4 (50%)	8 (57.1%)
Virologic and immunological endpoints			
Median HIV RNA VL at 6 months of age	5.4 (3.6–6)	5.7 (5.4–5.8)	5.3 (3.4–5.8)
Median ddPCR at 6 months of age, N = 32	7880 (3938–14,764)	8586 (3584–11,920)	7278 (3584–11,920)
Median ddPCR at 6–11 years of age, N = 35	144 (85–410)	92 (65–348)	358 (59–786)
Indeterminate/negative WB at 6–11 years of age	5 (33.3%)	3 (37.5%)	0 (0%)

Early refers to cART initiation up to 6 months of age, and late after 6 months.

Table S4. Lifetime ART regimens for study participants (N = 37). Switching to a second-line regimen is defined as changing at least one NRTI and changing the backbone drug class or changing the protease inhibitor.

Patient	Regimen	Age at First Treatment Initiation (months)	Start Date	Switched to Second-Line Regimen? [1]
1	D4T,3TC,LOP/r	13	17 October 2006	
	AZT,3TC,LOP/r		30 March 2010	
2	AZT,3TC,NVP	4	5 March 2009	Y
	AZT,3TC,LOP/r		18 March 2009	
	AZT,3TC,EFV		6 January 2015	
3	AZT,ddI,LOP/r	10	8 August 2006	
4	AZT,3TC,LOP/r	4	18 February 2009	
5	ABC,3TC,LOP/r	5	29 July 2008	
6	AZT,3TC,NVP	15	3 April 2007	Y
	AZT,3TC,LOP/r		3 May 2010	
7	ABC,3TC,LOP/r	24	27 January 2009	
8	AZT,3TC,LOP/r	5	19 February 2008	
9	AZT,3TC,LOP/r	13	5 April 2010	
10	3TC,ABC,LOP/r	2	7 April 2008	Y
	AZT,3TC,LOP/r		11 May 2010	
	AZT,3TC,EFV		18 May 2011	
11	AZT,3TC,LOP/r		17 January 2012	
12	AZT,3TC,LOP/r	5	23 January 2007	
13	AZT,ABC,3TC	26	11 July 2006	Y
	3TC,ABC,NVP		13 November 2007	
	AZT,3TC,LOP/r		17 June 2008	
14	AZT,3TC,LOP/r	7	16 August 2005	
	D4T,3TC,LOP/r		19 January 2009	
	AZT,3TC,LOP/r		19 March 2014	
15	AZT,3TC,LOP/r	3	29 January 2007	Y
	AZT,3TC,NVP		23 March 2007	
16	AZT,ddI,LOP/r	38	22 November 2010	
	AZT,3TC,LOP/r		26 November 2013	
	AZT,ABC,LOP/r		5 May 2014	
17	ABC,3TC,LOP/r	7	25 November 2008	
18	AZT,3TC,LOP/r	6	14 February 2006	Y
	AZT,3TC,NVP		30 October 2007	
	AZT,3TC,EFV		3 February 2009	
19	ABC,3TC,NVP	2	25 August 2009	Y
	ABC,3TC,LOP/r		16 March 2010	
	ABC,3TC,NVP		7 April 2010	
20	AZT,ddI,NFV	1	10 March 2006	Y
	AZT,ddI,LOP/r		3 May 2008	
	AZT,3TC,LOP/r		22 June 2011	
21	AZT,3TC,LOP/r	32	7 February 2012	
22	AZT,ddI,LOP/r	14	3 February 2006	
	AZT,3TC,LOP/r		8 March 2013	

Patient	Regimen	Age at First Treatment Initiation (months)	Start Date	Switched to Second-Line Regimen? [1]
23	AZT,ddI,LOP/r	8	20 December 2005	
24	AZT,3TC,LOP/r	75	9 December 2011	
25	AZT,3TC,LOP/r	57	7 February 2012	
26	AZT,3TC,NVP	14	2 October 2009	Y
	AZT,ddI,LOP/r		1 February 2012	
	AZT,3TC,LOP/r		23 May 2012	
27	ddI,D4T,LOP/r	11	9 June 2006	
	ddI,AZT,LOP/r		19 October 2011	
28	AZT,3TC,LOP/r	4	11 October 2007	
29	AZT,3TC,LOP/r	25	15 July 2009	
30	AZT,3TC,NVP	19	22 December 2009	
	ddI,3TC,NVP		29 January 2010	
	AZT,3TC,NVP		23 December 2010	
	AZT,3TC,EFV		1 July 2011	
	AZT,3TC,NVP		4 July 2011	
	AZT,3TC,LOP/r		2 September 2011	
31	AZT,3TC,LOP/r	63	2 May 2012	
32	AZT,3TC,LOP/r	8	31 October 2007	
33	AZT,3TC,LOP/r	5	22 July 2008	Y
	AZT,3TC,EFV		17 July 2013	
34	D4T,3TC,LOP/r	11	26 July 2006	Y
	3TC, AZT,Darunavir/r		7 January 2010	
35	AZT,3TC,LOP/r	7	19 December 2007	
36	AZT,3TC,LOP/r	8	1 November 2007	Y
	AZT,3TC,NVP		22 February 2008	
	3TC,TDF,LOP/r		26 May 2009	
37	AZT,3TC,NVP	5	8 July 2010	Y
	AZT,3TC,LOP/r		13 July 2011	

1. 6 of 19 switches were for drug availability reasons and not defined as actual ARV switches to a second-line regimen based on the definition above.

Table S5. Clinical and laboratory details for five children with sustained virologic response.

Sex	Infection Intrapartum or in Utero?	Age at ART Initiation (months)	Type of ART	No of Regimens	Median HIV VL at Time of ART Initiation	VL after 6–11 months of ART	ddPCR at 6 months	ddPCR at 6–11 years	WB at 6–11 years
F	Intrapartum	9	PI	1	2900	<40	15,363	109	Pos
F	In utero	3	PI	1	<40	<40	3806	105	Pos
M	In utero	23	PI	1	60,807	<40	14,166	242	Ind
M	In utero	3	PI	2	315,681	<40	Missing	61	Neg
F	Intrapartum	57	PI	1	58,089	<40	16,840	264	Pos

F: Female, M: Male, PI: Protease Inhibitor, VL: Virus load, ART: Antiretroviral, ddPCR: digital drop PCR, WB: Western blot, Pos: Positive, Ind: Indeterminate, Neg: Negative.
